# Wildlife reintroduction: considerations of habitat quality at the release site

**DOI:** 10.1186/1472-6785-6-5

**Published:** 2006-04-13

**Authors:** Susan M Cheyne

**Affiliations:** 1 Wildlife Research Group, Department of Anatomy, University of Cambridge, Cambridge, UK

## Abstract

**Background:**

Assessing the suitability of a habitat prior to the release of animals is vital. Proper assessment of the flora will allow reintroduction programmes to determine whether the area will be capable of supporting the released animals in the long-term. Here data are presented from an island in Central Kalimantan, Indonesia which has been used as a release site for agile gibbons (*Hylobates agilis albibarbis*) since January 2003.

**Results:**

Methods and results regarding fruit abundance, fruit productivity, tree density and diversity are presented. This information is then analysed in the context of the island's suitability to sustain released gibbons and without impact on the resident fauna. Based on the above ecological characteristics, the final carrying capacity of the island is estimated to be between 3 and 19 gibbons.

**Conclusion:**

These data highlight the need to survey areas being considered for release of gibbon prior to the release taking place. For reintroductions to be successful, long-term habitat assessment is vital, both pre- and post-release.

## Background

The principal objectives of a reintroduction project are to establish a viable, free-ranging population in the wild, of a species, which has become globally or locally extinct in the wild [1]. Within this reintroduction concept there are two types of reintroduction: (a) **re-establishment: **the use of captive-bred animals to re-establish an extinct population and (b) **stocking reintroduction **which involves supplementing a declining population with captive-bred animals [2]. I propose a third definition: 'population reintroduction' which should refer to the use of wild-born, captive-raised animals to re-establish a population where it has become locally extinct, but only if the area can be adequately protected. Reintroduction addresses conservation on two levels: (1) animals kept illegally as pets are rescued, rehabilitated and returned to the wild, thereby addressing the illegal trade in wild animals and the welfare of these animals while in captivity and (2) by reintroducing animals into areas where they are locally extinct, the wild populations are supplemented and additional areas of forest can be protected. Reintroduction may be the only viable way of repopulating areas of forest that have been devastated by hunting.

For rehabilitation to succeed, equal care and planning should go into both the pre-release and post-release phases. Past experience has identified several factors that affect the success of the release of previously held captive animals: negative impact on the native flora and fauna (chimpanzees and orang-utans, [3]), mortality due to animals being unused to natural predators in the release site (golden lion tamarins, [4]), poaching, traffic, shooting by humans (drills, [5]), inter- and intra-specific competition (gibbons, [6]), and poor habitat quality at the release site (gibbons, pers. obs.).

With post-release, not only must the animal's behaviour be monitored, but also the habitat of the release site must be surveyed adequately. Providing that a detailed habitat survey is carried out prior to the release, there should be limited impact on the native flora and fauna by gibbons. Post-release monitoring of forest as well as individuals must be as comprehensive as possible and should follow established scientific data collection methods. The result of poorly planned releases and reintroduction of primates has clear results: failure of the primates to adapt to the wild, failure of the population to increase and negligible conservation impact.

Here I present data from the habitat survey of an island where a pair of captive-raised adult *Hylobates agilis albibarbis *were released in January 2003 from the Kalaweit Gibbon Rehabilitation Project in Central Kalimantan. The habitat analysis was carried out to ensure that the island was capable of supporting the gibbons and the resident wildlife i.e "can Mintin Island sustain a population of gibbons, and if so, how large a population?"

## Results and discussion

Estimating density of fruit trees by simply dividing the number of trees by the area of the island ignores the between-species differences in the size distribution of adult trees and the intensity of production activities, thus biasing the result. To avoid this, [7] have developed a productivity index:

I_m _= F_km _b_k _d_k_

Where Fm = the fruiting score of all sampled individuals in species 'k' during month 'm'.

b_k _= mean dbh (diameter at breast height) of any adult tree for species 'k'

d_k _= density (number per ha) of adult trees for species 'k'

Diversity of fruiting trees was estimated using the Shannon-Weaver index and the frequency of each species in a block was compared to a Poisson distribution as a check for the random distribution of the species. Shannon-Weaver (based on a formula in [7]):

H = - pi (ln(pi))

where pi is the relative frequency of a species in a given sample.

The Shannon-Weaver index measures species richness. If only a few species account for most of the biomass, then the Shannon-Weaver index number is low. A reading of 2.0 would be a rich, diverse plant community. S-W Index values (H) can range from 0 to ~4.6 using the natural log (versus log10). A value near 0 would indicate that every species in the sample is the same. Conversely, a value near 4.6 would indicate that the numbers of individuals are evenly distributed between all the species present.

Mather (1992b) [8] compared several areas of Borneo and Peninsular Malaysia for relationships with their density and distribution. Based on this, I used Mathers's data for comparative purposes. By extrapolating data from Mather's sites which had gibbons, an estimate for potential numbers of gibbons/km^2 ^on Mintin Island was calculated based on food tree and dipterocarp availability (Table 3), to determine the potential carrying capacity. By extrapolating from the other sites, an estimate for potential numbers of gibbons/km^2 ^on Mintin Island was calculated. This was to determine the potential carrying capacity of the island based on the keystone species of figs [9]. Much of the data from other sites were based on one variable only (fig density, dipterocarp density) and this was used to create a best-fit for Mintin Island. Figs are keystone species due to their asynchronous fruiting pattern [10-13] i.e. all trees do not all fruit at the same time, thus there are always some fig trees in fruit in the forest, making this genus an important fall-back food for gibbons in times of food shortage (Cheyne, unpublished data). Dipterocarps are important for gibbons for singing locations, morning calling is from dipterocarp tress in 74% of singing bouts (n = 1076; [14,15]. Dipterocarps are rarely primate feeding trees (except for leaves, [16], Cheyne, unpublished data) and their height is important for gibbons to broadcast their morning chorus and duet.

### Fruit abundance

Direct observation of the canopy is the simplest and most convenient method in open and low forest, but problems arise when the method is employed in dense, tropical forest where emergent trees are often over 50 m high ([17]). This was not a problem on Mintin Island, where the forest is maturing secondary vegetation with a maximum tree crown height of 23 m.

Based on Mather's [16] index, 17.1% of the trees on Mintin are in the 10 most important gibbon food genera (Table 2), and the percentage of may be a valuable index for comparing potential food productivity between sites. Results for Mintin, show varied levels of productivity for the species/genera, perhaps because Mintin is an island of recovering secondary forest and may have more varied and less predictable productivity than pristine forest [18].

Adaptation to life in the forest presents many obstacles for the gibbons and one of these should not be excessive food competition through poor management of the islands resources. This method does have limitations: the extrapolation only deals with data on gibbon densities based on the percentage of trees in the important food genera for gibbons and does not account for other fauna. E.g. Barito Ulu does not have orang-utans or large groups of macaques, whereas Mintin does have macaque groups. Thus, at Barito Ulu, gibbons may occur at higher densities, because there is less competition from other species. Other fauna e.g. macaques and hornbills will need figs when other fruit sources become scarce, so, competition from these animals may reduce the fruit available for gibbons and render my estimates too high. The relationship between the four sites is not strong, as evident by the result of the regression. To counter this I present a 95% confidence interval range for the number of gibbons that could be supported on Mintin i.e. the range at which the estimated mean value from the regression analysis could lie.

### Fruit productivity

Flower production was lowest during the dry season from June to August 2003 and young leaf availability increased in the lead up to the dry season. Fruit production was erratic, and would probably result in the gibbons having to feed on trees not on Mather's list. This list does contain 40 genera on which gibbons feed, thus flexible diet allows them to adapt to food shortages.

### Density

A regression analysis was carried out on the available data for gibbon group size compared to the density of figs (R^2 ^= 54.3 p = 0.015, n = 9). Figs comprised the main fruit source for two months during this study (January-February 2003) as there was very low availability of other sources of food e.g. non-fig fruit. As a result, figs are likely to be an important fruit source for the other frugivores on the island and there may be competition in times of scarcity.

Dipterocarps are not common food sources for gibbons but they are used for sleeping and singing. Density was calculated as the number of trees/hectare. Mintin Island also supports a density of 1.8 dipterocarp trees/ha, which are important for providing sleeping and singing platforms for the gibbons (Table 3).

Mintin Island is placed into Table 4 to show the expected group size and number of groups that it could support. The fig data appear to be very variable, hence the large confidence intervals.

### Diversity

Mintin Island has a Shannon-Weaver H-value of 2.98 indicating that the island hosts a rich and diverse plant and tree community. Pristine forest is inevitably going to support a higher diversity than logged, recovering forest but this indicates that the diversity of Mintin Island is not quite species rich and diverse, and is probably capable of supporting a similar diversity of fauna, albeit at lower densities.

### Suitability of Mintin as a release site

Based on preliminary data (Table 5), Mintin Island should be able to support at least two pairs of gibbon with a total carrying capacity of 2.5–20 gibbons/km^2 ^and group sizes of 1.5–3.5 individuals. The island should reach carrying capacity no later than the F_2 _generation, thus the offspring will need to be translocated to a larger site. This area has been identified as a 1500 ha forest near the Kalaweit Gibbon Sanctuary in Central Kalimantan.

[19] showed that there was a positive correlation between dipterocarp abundance and the biomass of gibbons a forest can support. Dipterocarps are an important structural component of the forest for gibbons as they provide platforms for sleeping and singing. There are few large dipterocarp trees left on Mintin, which, combined with the small area of the island, means that Mintin probably cannot support more than two groups, if dipterocarp density is indeed a limiting factor for the Mintin gibbons. It is possible that the lack of dipterocarps was a factor in explaining why the gibbons did not sing very often, though I believe that the lack of singing is more likely due to the fact that the pair was in very unfamiliar territory and that they separated immediately after release. When the gibbons did start to sing, they sang from dipterocarp trees (n = 6).

Most tropical, woody plants tend to produce new leaves and flowers in bursts rather than continuously, and there are considerable seasonal variation in the abundance of these food sources [20]. This variation affects the abundance of primary consumers (e.g. frugivores and folivores) that will also have to modify their behaviours accordingly. If the year 2003 was a bad fruit year it is possible that there will be a bigger influx of animals to the island in better years, thereby increasing food competition with the gibbons. On the other hand, there may also be more food available if 2002/03 was lower than normal for fruit production, thus mitigating the effects of food competition. Clearly data are needed to determine exact levels of competition between the gibbons and other wildlife for the island's resources. This is a post-release issue and herein I discuss the need for detailed pre-release habitat analysis.

The island also supports groups of macaques and proboscis monkeys. Both these species are transient and were observed to swim from the island to the mainland (10 sightings of macaques in two groups and 8 sightings of proboscis monkeys in one group). They were observed on the island throughout the eight months that the gibbons were present, though not every day; thus, the macaques and proboscis monkeys can be considered as semi-permanent residents of Mintin Island. It is very important to collect data on the resource use by the macaques (*Macaca nemestrina *and *M. fascicularis*) and proboscis monkeys to effectively predict the resource availability for gibbons in the same area. Hornbills, being frugivorous, could be a potential competitor for gibbons given the limited production of fruit across the year. Therefore, feeding requirements of hornbills for limited fruit trees need to be identified and quantified for determining long term survival of the released gibbon populations in the proposed release site. Ideally, feeding rates can be collected on the released gibbons and the wild macaques to obtain data on food intake at different times of year and to observe how much overlap in diet there is between the two species. These data have to be collected post-release, and will be of great importance in the final phase of release, i.e. gibbons reintroduced to contiguous forest. This study does not include data on feeding rates and food intake, as the purpose is to address the issue of habitat quality assessment pre- and post-release. Feeding rates, food competition and food intake will form part of the post-release monitoring of gibbons due for reintroduction in 2007.

## Conclusion

Tropical forests continue to disappear at a phenomenal rate and the illegal pet trade continues with no sign of abating any time soon. The numbers of gibbons being kept in captivity will only increase as their forest homes are opened up for plantations, logging concessions and for access. Rehabilitation can work in conjunction with habitat protection in terms of protecting areas for reintroduction and establishing rehabilitation training centres where there are already wild gibbons. Rehabilitation and reintroduction is becoming the only viable option to save the hundreds, possibly thousands of pet gibbons all over the world and to repopulate the large tracts of forest which no longer have gibbon populations due to hunting.

In the IUCN/SSC Reintroduction Specialist Group: Guidelines for Non-human Primate Reintroductions [21] it is stated "reintroductions should only take place when the taxon's habitat requirements are satisfied and likely to be sustainable for the foreseeable future. If the taxon's basic habitat and ecological requirements cannot be determined, the animals should not be released." The only way to meet these requirements is to conduct habitat analysis of the release site, both pre- and post-release. Here I have shown that the release island of Mintin has the capacity to support at least three groups with about 5 gibbons/group (15 individuals). Although extrapolations of gibbon food trees estimates the maximum population size to be 19 individuals, there is competition from other animals, which is likely to prevent Mintin supporting four gibbon groups. The accurate analysis of the release area is essential if the released animals are going to survive in the future and for them to become nutritionally independent as soon as possible post-release. I recognise that gibbons released onto an island will not be viable for establishing a long-term population as the island cannot sustain a large population indefinitely. Nevertheless, it is important that releases of animals are carried out in a manner that allows post-release data to be collected. Using the island as a half-way house for the released gibbons allows us to assess their adaptation and identify any problems. A half-way house is an area of good quality forest were reintroduced animals are released immediately following the rehabilitation period in the cage [22]. The half-way house allows the released animals to adapt to the forest while remaining under the care and supervision of project staff i.e. the animals will be monitored to observe how they are adapting to the forest. The half-way house is generally a small, isolated area of forest where the animals are free to roam but where they can still be located if there are any problems. Due to the lack of accurate and reliable data on the behaviour of released gibbons it is essential that all stages of the rehabilitation and release process are monitored. It is not known how gibbons will adapt to the forest without a half-way house experience, the purpose of the half-way house is to identify any problems quickly and to ensure all animals are adapting well to the wild. As long as there are limited data available on the behaviour of reintroduced gibbons, the slow approach using a half-way house is by far the sensible. Without information from the animals' behaviour in a half-way house setting, we risk releasing unprepared animals who will be difficult to follow and monitor. Using this study as a template, future releases can take place in contiguous forest. This is a preliminary study based on available data and one of the first of it's kind to attempt to address issues of habitat suitability at release sites. This study looked at the important aspect of the relationship seen between gibbon population density and floristic composition. I am aware that predator densities, the densities of other frugivore competitors, human impacts, demographic stochasticity in births/deaths are important aspects but are not variables ones that can be reasonably studied for an initial assessment of a release area. These variables should form the basis of the long-term post-release monitoring of the release site and the impact of the gibbons on the island.

## Methods

**Figure 1:**
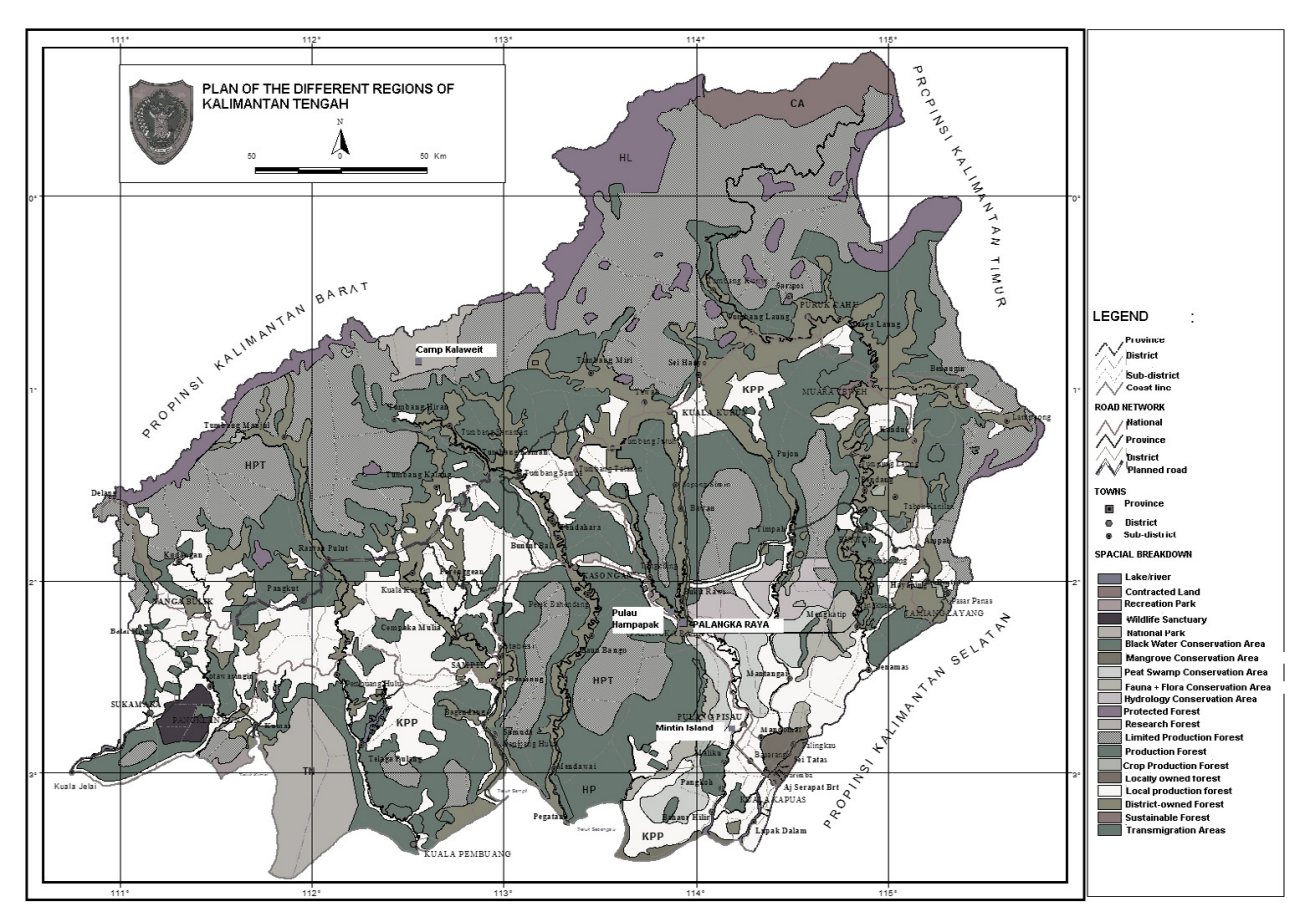
Map of Central Kalimantan showing location of Mintin Island.

**Figure 2:**
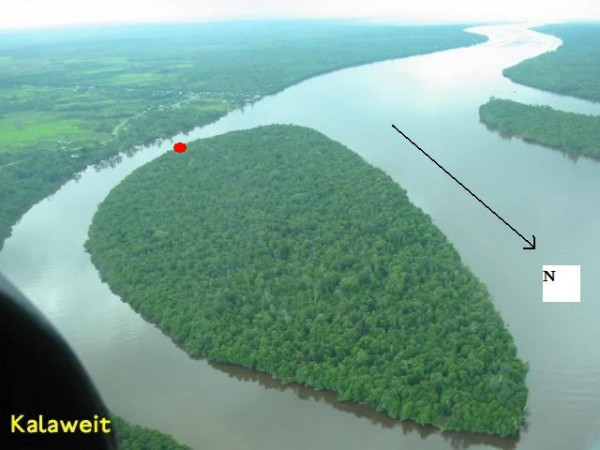
Arial photo of the release island showing release site (star, photo courtesy of Chanee, Kalaweit Director).

The release site is an island of 100 ha situated on the Kapuas River, 75.4 m south of Palangka Raya on the road to Banjarmasin in Central Kalimantan, Indonesian Borneo (Figure 1), known as Pulau Mintin (02° 50' N, 114° 12' E, Figure 2).

It is lowland freshwater swamp forest (FWS) at an altitude ranging from 10 to 22 m a.s.l. and is frequently flooded by the seasonal rains (the surrounding area is a floodplain), making the floor unsuitable for the gibbons and encouraging them to remain in the treetops. The island is 100 ha of regenerating secondary forest in the Kapuas River. This island is also home to wild *Nasalis larvatus *(group size 8), *Macaca nemestrina *(group size 9–13), *M. fascicularis *(group size 5–10) and several hornbills (exact numbers unavailable). The macaques and proboscis monkeys were not observed on the island continuously and were seen to swim between the island and the mainland (a distance of about 200 m). There are no large carnivorous predators on the island though crocodiles were seen in the interior of the island during the wet season. There are reports of large snakes on the island though the author did not encounter any during this study. The purpose of this paper is to highlight the need to assess habitat suitability and quality before any release takes place, and to stress the need for ongoing monitoring of habitat quality post-release. To assess the feeding competition between reintroduced gibbons and other species, data are needed on food competition, feeding rates and food intake. These data form the post-release monitoring phase and can only take place once an area has been deemed suitable for reintroducing gibbons. Feeding competition data are not available as the macaques were not habituated. Analysis of the activity budgets of the released gibbons shows them to be spending 37% of their time feeding. This figure is equivalent to other studies of wild agile gibbons [23,24].

The local community were accustomed to hunting and logging the island and many logging skids are still present. Following negotiations with local chiefs and elders the local people have agreed to stop logging and hunting on the island and are actively protecting it, in collaboration with the local police.

To determine the suitability of the habitat for the pair of Kalaweit gibbons it was necessary to establish the fruit abundance and productivity of the release area, Mintin Island. From September 2002-December 2003 a preliminary survey of known fruiting trees on the release island that were eaten by macaques and/or gibbons was carried out by the local people of Mintin Village, based on their knowledge of fruiting trees on the island. This initial survey gave local names only. With the assistance of a botanist from Palangka Raya University, SMC collected samples of all fruiting trees for which the Indonesian or scientific names could not be identified through local name alone. Scientific identification was made by Erna Shinta, resident botanist at CIMTROP (Centre for International Co-operation in Management of Tropical Peatlands) at Palangka Raya University. Trails were not cut on the release island, nor were transects, but every fruit-producing tree was counted in the initial survey by dividing the island into three block (based on average canopy height). The island is 100 ha and subsequent surveys noted the number of marked trees that were producing fruit for each species in each block. This number was then extrapolated using the list of total tree numbers to obtain abundance.

In the initial floristic survey circa 6000 trees were examined. Fruit-producing trees >10 cm Dbh were marked during the initial habitat survey. Due to past logging, the number of large trees (>20 Dbh) were fewer then would be found in pristine forest. All fruit trees on the island were counted and identified and density calculated (Table 1). Fruit trees identified as the most important for gibbons (Table 2) and >10 cm Dbh (diameter at breast height) on Mintin Island were surveyed twice a month (for one year). The number of genera that were producing young leaves, flowers and ripe fruit. Trees were scored for fruiting or not, presence of flowers or young leaves. Based on the proportion of those producing fruit each month from each species, a value for overall productivity for that species was extrapolated.

## Authors' contributions

SMC conceived of the study and carried out the behaviour and ecological habitat analysis for this research.

## Figures and Tables

**Table 1:** Main fruit tree density and numbers.

Family	Genus/species	Number of trees	Density (trees/ha)
Guttifferae	*Garcinia bancana*	303	3.03
Moraceae	*Ficus benjamina*	106	2.63
Anacardiaceae	*Gluta renghas*	125	2.56
Meliaceae	*Aglaia simplicifolia*	99	3.10
Rubiaceae	*Ixora sp*	96	4.97
Euphorbiaceae	*Baccaurea spp*	360	2.67
Guttifferae	*Knema laurina*	123	1.48
Myrtaceae	*Eugenia spp*	198	1.98
Moraceae	*Ficus spp*	76	1.06
Annonaceae	*Polyalthia sp*	233	2.33
Guttifferae	*Callophyllum rhizophorum*	111	4.66
Moraceae	*Artocarpus sp*	238	2.91
Myristicaceae	*Gymnacranthera sp*	53	2.98
Anacardiaceae	*Mangifera foetida*	228	2.80
Flaucourtiaceae	*Flacourtia rukam*	189	2.32
Polygalaceae	*Xanthophyllum sp*	241	2.41
Mimosaceae	*Parkia sp*	107	3.25
Dillenaceae	*Dillenia reticulata*	290	1.09
		3176	

**Table 2:** Productivity of 10 most important gibbon food trees (as identified by Mather,1992).

Family	Genus/species	TOTAL	Productivity index
Guttifferae	*Garcinia bancana*	303	3.03
Moraceae	*Ficus benjamina*	106	3.69
Euphorbiaceae	*Baccaurea *sp	360	2.67
Guttifferae	*Callophyllum hosei*	148	6.14
Myrtaceae	*Eugenia *sp	198	1.98
Annonaceae	*Polyalthia *sp	233	2.33
Moraceae	*Artocarpus *sp	238	2.91
Anacardiaceae	*Mangifera foetida*	228	2.80
Polygalaceae	*Xanthophyllum *sp	241	2.41
Dillenaceae	*Dillenia reticulata*	290	1.09

**Table 3:** Gibbon density and percentage of important food trees and dipterocarps.

Site	% of trees in important species list	Gibbons/km^2^	% Dipterocarps	Gibbons/km^2^
Sepilok^1^	9.4	5.5	NA	NA
Kuala Lompat^2^	10.3	6 (or 8.4)	1.0	6.0 (or 8.4)
Mintin Island	17.1	5–10 (estimated)	1.8	5.9–19.4 (estimated)
Pasoh^3^	12.1	8.4	NA	NA
Barito Ulu^4^	18.3	10.5	42.6	18.3

Data sources: 1 = Davis and Payne 1982, cited in Mather (1992); 2 and 3 = Marsh and Wilson cited in Mather (1992); 4 = Mather (1992).

**Table 4:** Density of gibbons based on fig tree density (adapted from Mather, 1992) with an estimated sustainable group size and number of groups that can be supported on Mintin Island.

Site	Figs/ha	Group Size	Groups/km^2^
Sepilok, Sabah	0	1.5	2.7
Tanjung Puting, Kalimantan	1.0	2.9	3.0
Siberut, Mentawi Islands	1.3	2.1	3.7
Sungai Sakam, West Malaysia	2.0	2.5	3.3
Danum, Sabah	2.3	2.1	3.5
Mintin Island, Kalimantan	3.0	1.5 – 3.5 (estimated)	2.5 – 4.5 (estimated)
Pasoh, West Malaysia	4.0	2.1	4.0
Barito Ulu, Kalimantan	6.0	3.6	2.8
Kutai, East Kalimantan	6.6	3.6	4.0
Kuala Lompat, West Malaysia	8.0	4.1	4.0
Ketambe, North Sumatera	27.0	4.3	4.5

**Table 5:** Summary of gibbon density based on 3 main vegetation characteristics.

Variable	Estimated Gibbons/km^2^
% of Dipterocarps	5.9–19.4
Density of fig trees	3.75–15.75
% of trees in 10 important gibbon food genera	5–10
